# ASSESSING THE CAREGIVING NEEDS OF ASIAN AMERICAN AND PACIFIC ISLANDER ADRD CARE PARTNERS

**DOI:** 10.1093/geroni/igae098.0934

**Published:** 2024-12-31

**Authors:** Jeanine Yonashiro-Cho, Laura Mosqueda, Zachary Gassoumis

**Affiliations:** University of Southern California Keck School of Medicine, Los Angeles, California, United States; University of Southern California Keck School of Medicine, Pasadena, California, United States; University of Southern California, Los Angeles, California, United States

## Abstract

Care partners (CP) are essential in supporting persons living with dementia (PLWD). Asian American and Pacific Islanders (AAPI) may be more likely to engage in caregiving and may cope with caregiving stressors in culturally-distinct ways, thus necessitating development of culturally-appropriate supports and interventions. This study explores CP needs and identifies potential targets for intervention among AAPI communities. Explanatory sequential mixed-methods analyses were conducted with AAPI dementia care partners (N=8) who took part in the Better Together Dementia Care study. Caregiving characteristics and outcomes data from quantitative surveys were paired with findings from a thematic content analysis of semi-structured interview data. Key informant interviews AAPI CPs and providers will also be analyzed. Participants included AAPI spousal (n=4) and adult child (n=4) CPs supporting a PLWD, including 4 who had recently experienced elder mistreatment. Due to their emigration (n=3), spousal caregivers reported a lack of instrumental caregiving support from extended family. In child-parent dyads, PLWDs had lost their partners due to death (n=3) or divorce (n=1). PLWDs varied in relational and emotional closeness with children. Preliminary findings highlight the diversity of CP experiences. Romantic partners may serve as the preferred “default” CP; in their absence, adult children may relied upon more heavily. While traditional API cultures often emphasize a child’s duty to care for elders, acculturated older adults may prefer to receive care from their partner whenever possible. Findings also suggest immigrant CPs and CP-PLWD dyads may benefit from culturally-specific instrumental supports to supplement the emotional support provided from family members overseas.

